# Economic results of a palivizumab seasonal prophylaxis using a cohorting software and vial sharing

**DOI:** 10.1186/1824-7288-36-48

**Published:** 2010-07-07

**Authors:** Elio Coletta, Salvatore Coppolino, Febronia Federico, Francesco Fulia

**Affiliations:** 1 U.O.C. Pediatria e UTIN, P.O. "Barone I. Romeo", Via Mazzini, Patti, Messina (98066), Italy; 2 Dipartimento del Farmaco, Cittadella della Salute, Viale Giostra, Messina (98100), Italy; 3 Servizio di Farmacia, P.O. "Barone I. Romeo", Via Mazzini, Patti, Messina (98066), Italy

## Abstract

**Background:**

Respiratory syncytial virus is the most important pathogen in lower respiratory tract infection in infants and young children. In high-risk populations it may develop severe, sometimes fatal, lower respiratory tract infections. A proportion of these infants require admission to intensive care units due to the severity of the condition and the level of care needed. Furthermore, we must consider the possible increased risk of asthma following RSV infection in infancy.

**Methods:**

The aim of this work is to show how we strictly coordinated, during the 2008-2009 RSV season, the delivery of prophylaxis while minimising drug cost through vial sharing and cohorting infants with a software performed through Visual Basic programming system.

**Results:**

By using this method we have been able to obtain a saving of the 29.2% compared to the theoretical amount. No infant requested hospitalisation for a RSV infection.

**Conclusions:**

Such a model ensures all patients to receive appropriate immunization and thus positively influencing the cost-benefit of palivizumab prophylaxis. We hope that our model of care delivery will be of use to other hospitals.

## Introduction

Respiratory syncytial virus (RSV) is the most important pathogen in lower respiratory tract infection in infants and young children [1]. It causes coughs and colds in winter season. The virus belongs to the same family as the human parainfluenza viruses and mumps and measles viruses. By 2 years of age, approximately 80% to 90% of children experience at least one episode of RSV infection. Although the majority of RSV infections are mild, high-risk populations such as premature infants (gestational age < 33 weeks) or children with hemodynamically significant heart disease or with lung abnormalities or with immunodeficiency may develop severe, and sometimes fatal, lower respiratory tract infections [2]. In Italy, about 4-5000 RSV infected high-risk infants are hospitalized every year. A proportion of these infants require admission to intensive care units due to the severity of the condition and the level of care needed [3] and have higher mortality rates than healthy infants.

Furthermore, as potential long-term sequelae, we must consider the possible increased risk of asthma and allergies following RSV infection in infancy and its impact on life quality [4].

Palivizumab, an intramuscular humanized mouse monoclonal antibody, is used to reduce the risk of hospitalization secondary to RSV infection [5]. Seasonal prophylaxis with this antibody demonstrated clinical efficacy and satisfactory tolerability and it doesn't interfere with the administration of other vaccines [6,7].

The aim of this work is to show how we strictly coordinated, during the 2008-2009 RSV season, the delivery of prophylaxis while minimising drug cost through vial sharing.

## Materials and Methods

The 2008-2009 RSV prophylaxis started in November 2008 and ended in April 2009. The vaccination program was designed to ensure that every eligible infant received RSV prophylaxis and his or her parents received necessary education to prevent RSV-related hospitalisation. The 4 bed UTIN unit at "Barone I. Romeo" Hospital, Patti (Messina) accepts 249 admissions per year. During the RSV prophylaxis season to 24 high-risk eligible children was administred the prophylaxis with palivizumab. High-risk criteria indicating the prophylaxis are reported in Table 1. The current recommended palivizumab dosage is 15 mg/kg intramuscular injections (once per month for a total of 5-6 doses during the RSV season). The cost of 50 mg and 100 mg vials of Synagis^® ^(Abbott Laboratories Limited) were 490.37€ and 814.35€ respectively. Synagis requires storage in a refrigerator (2 to 8°C) and once reconstituted, the palivizumab shelf life is estimated at six hours [8] and multidose use of single-use vials is proven safe [9].

**Table 1:** High-risk criteria

Evidence grade I:	Infants born from 32 weeks of gestation or earlier to 12 months at the beginning of RSV season.
Evidence grade I:	Infants and children younger than 24 months with CLD who required medical therapy (supplemental oxygen and/or drugs).

Evidence grade I:	24 months old or younger children receiving medication to control hemodybamically significant heart disease or diagnosed with moderate to severe pulmonary hypertension or diagnosed with cyanotic heart disease.

Evidence grade III:	Infants, born at 32 to less 35 weeks of gestation, who are 12 months old, or younger, at the start of RSV season with at least two of the following risk factors: low weight at birth (<2.5 Kg), exposure to environmental air pollutants or tobacco smoke, lack of breast-feed, twin birth, chest malformation, hematologic diseases, cystic fibrosis, school-aged siblings, congenital abnormalities of the airways, cancer, severe neuromuscular diseases, immunodefiency or living where the access to a hospital is difficult.

We used a collaborative framework for the delivery of RSV prophylaxis. The multidisciplinary team (pharmacists, physicians, nurses) collaborated to create a RSV prophylaxis program logic model, ensuring that each discipline's perspective of the program process was considered. For each program component, the team identified process and program objectives and outcomes.

Before the beginning of the prophylaxis all infants were visited and weighted and the obtained data were recorded on a database. In order to start the administration infants were grouped in four cohorts of five and one of four with a software performed by Coppolino S. through Visual Basic programming system. Visual basic is used to write Windows-based computer programs; by doing so you are not bound by the limitations of a particular "off-the shell" computer program. What is more you are able to design applications to meet your own specific needs [10].

**Figure 1:**
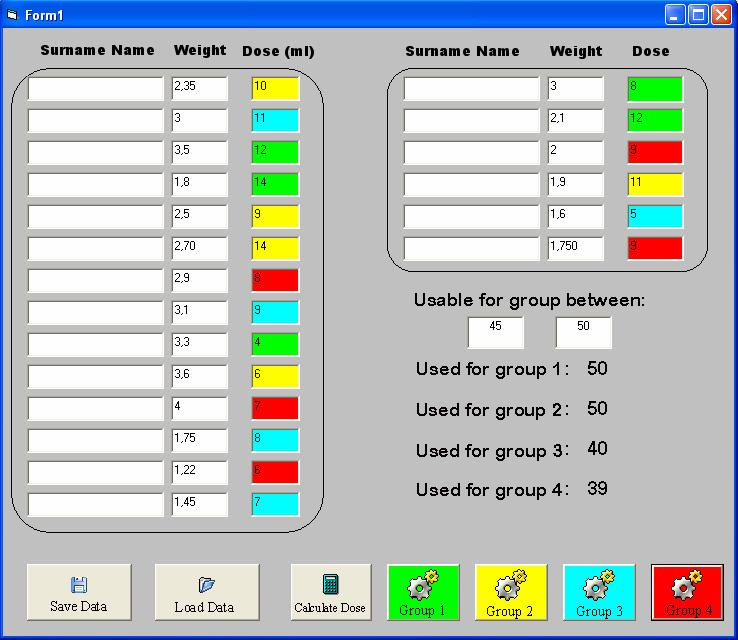
**screen of the developed software**. This figure shows the main screen of the software used to schedule young patients during treatment It automatically evaluated the amount of drug to be administered and then it sorts infants by weight suggesting the best way to minimize waste.

This software requests only to insert infants names and their weight. By the clicking of a button the software calculates the palivizumab dosage, in mg, to be administrated to each infant, and following to the vial selected (50 mg or 100 mg one) automatically divide infants in groups, evidenced by different colours, to use as few vials as possible to minimise waste (Fig. 1). Children marked with the same colour were scheduled to be vaccinated within the same day.

All data were automatically collected in a ".txt" file created to generate reports to be analysed afterwards with common softwares.

For each dose, infants attended the Ambulatory Ward on five consecutive afternoons. The second dose was administred after 3 weeks and subsequent ones at 4 weekly intervals. In total 6800 mg were bought and 6200 mg were given to patients. During the season, adverse events following immunization did not occur.

## Results

All infants successfully completed their full course of RSV prophylaxis and were followed for 150 days after the last scheduled injection. No one requested hospitalisation for RSV infection.

We calculated theoretical vial usage if every infant had been individually dosed with one vial and compared this with our real use, obtained by using the cohorting software and vial sharing. The aggregate seasonal drug cost for the season was 56.706,92€ instead of 80.087.19€ with a saving of 23.380,17€ (29.2% of theoretical amount). At the individual partecipant level the average seasonal palivizumab prophylaxis cost was 2372,69€. All data are reported in Table 2.

**Table 2:** Use of palivizumab and resources saving during 2008-2009 campaign

	Theoretical single use	Real use with vial sharing	Saving
50 mg vials	67	16	51

100 mg vials	58	60	-2

mg wasted	3.176,88	777,08	2.399,80

Value	80.087,09€	56.706,92€	23.380,17€

## Discussion

During all past seasonal prophylaxis with palivizumab we treated an almost constant number of high-risk eligible children for every year.

By using vial sharing and the above described software we obtained a drug cost saving of 25% compared to 2007-2008 season. Regarding the 2009-2010 campaign, in which we used vial sharing and software again, the cost saving was of about 2%, more or less the same of 2008-2009 season and linked to the children weight.

There are main other considerations besides costs in clinical decision making, but the careful use of resources must always be considerated.

In-hospital interdisciplinary communication and working relationships were a program strength point, particularly the relationship between pharmacy and " Pediatria e UTIN" staff. This strength was attributed to the ongoing opportunity for dialogue.

Like any expensive healthcare intervention, palivizumab immunization must be used judiciously. Our experience shows that it is possible to minimise the cost by an accurate cohorting and by multidose distribution with a maximising use of 100 mg vials in preference to the more expensive 50 mg vials for cost saving with no increased risk to patients. This does, however, required tight coordination between hospital pharmacist and ward and patient selection to discard ineligible children.

## Conclusions

The use of palivizumab can be optimized through a model in which children are prospectively identified and vials are shared. Such a model ensures all patients to receive appropriate immunization and thus positively influencing the cost-benefit of palivizumab prophylaxis. We hope that our model of care delivery to high-risk infants will be of use to other hospitals who seek to optimise delivery of their RSV immunization programs.

## Competing interests

The authors declare that they have no competing interests.

## Authors' contributions

All the authors contributed in the conceiving, design and realization of the manuscript. All authors read and approved the final manuscript.
